# Brain abscess and periodontal pathogens (*Fusobacterium Nucleatum*). Report of a case

**DOI:** 10.1002/ccr3.3173

**Published:** 2020-08-02

**Authors:** Debora Franceschi, Valentina Giuliani, Veronica Giuntini, Giovanpaolo Pini Prato

**Affiliations:** ^1^ Department of Experimental and Clinical Medicine University of Florence Florence Italy

**Keywords:** brain abscess, *Fusobacterium Nucleatum*, periodontal pathogens

## Abstract

Patients who develop brain abscesses must be evaluated through a complete set of diagnostic tests including a microbiological and clinical periodontal assessment. A genetic comparison of the pathogens from intracranial/extracranial sites is necessary.

## Graphical abstract

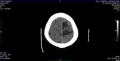

**Clinical relevance**

**Scientific rationale for the study**: Patients who develop brain abscesses must be evaluated through a complete set of diagnostic tests including a microbiological and clinical periodontal assessment.

**Principal finding**: A brain abscess caused by *Fusobacterium Nucleatum* occurred after a professional oral hygiene session. The microbiological finding of the periodontal pockets indicated the presence of *F Nucleatum*, previously isolated in the brain abscess samples.

**Practical implications**: A genetic comparison of the pathogens from intracranial/extracranial sites is necessary to verify the cause/effect relationship. Patients reporting systemic infection caused by periodontal pathogens must follow an appropriate periodontal therapy and stringent protocol of oral hygiene maintenance.

## 1 INTRODUCTION

The brain abscess is a rare but severe infection that can lead to death. The diagnosis is difficult and is usually made on the basis of clinical symptoms and instrumental investigations, such as computerized tomography (CT scan) or magnetic resonance imaging (MRI). Brain abscesses are most frequently caused by bacterial dissemination from a primary lesion at a distant site.^1^ One important site of primary infection can be the oral cavity.^2^

The primary infection located in the oral cavity is of paramount importance; in fact, a feature that is unique to the oral bacterial biofilm, particularly subgingival plaque biofilm, is its close proximity to highly vascularized tissues. A recent review investigating the pathogenesis, microbiology, interventions and outcomes of brain abscess of oral origin indicated four possible routes of odontogenic spread: (a) hematogenous systemic bacteria; (b) direct venous drainage via the facial and pterygoid vein system to the cavernous sinus; (c) inoculation via contiguous extension or introduction by foreign objects; (d) lymphatic drainage.^2^

Consequently, it has been assumed that the patients with periodontal disease may have a much higher risk of developing bacteremia from daily oral activities (B‐EOA) and, hence, systemic diseases of oral origin.^3^, ^4^, ^5^, ^6^, ^7^ Bacteremia of periodontal origin may be associated with many systemic diseases such as endocarditis, meningitis and subdural empyema, atherosclerotic vascular disease, and brain abscess.^8^, ^9^, ^10^

A rare but extremely severe complication of bacteremia of oral origin is the brain abscess, which is a life‐threatening infection that requires immediate neurosurgical attention. The incidence is between one and eight per 100 000 patients per year in the USA.^11^ On this issue, the literature consists on few individual case reports showing brain abscess “associated with” primary oral infection, however, a clear relationship between pathogens found in the brain abscess and those of oral origin is lacking.

The aim of this case report was to describe the case of a young patient with a brain abscess caused by *Fusobacterium Nucleatum*, associated with presence of FN in periodontal pockets and treated with active periodontal therapy and followed up for a long‐term period (7 years). A critical review of the literature was also performed.

## 2 CASE REPORT

**Figure 1:**
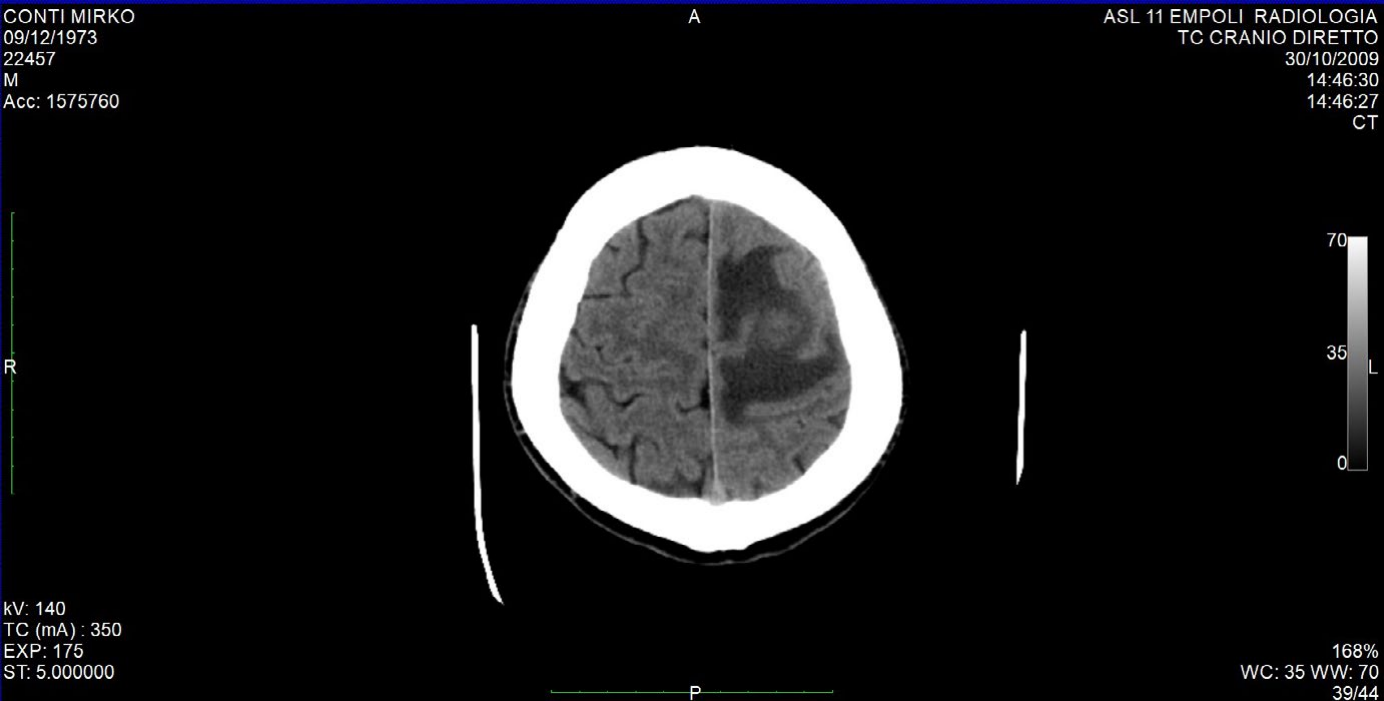
Computerized tomography: inhomogeneous isointense central portion compatible with abscess collection, surrounded by extensive hypodense area, to refer to perilesional edema

In October 2009, a 36‐year‐old healthy male presented to the Department of Internal Medicine at the Hospital of Empoli, Italy, with right‐side hemiparesis and other neurological symptoms such as impaired memory and unstable gait. Brain tomography (Figure 1) revealed a left central lesion suggestive of an intracranial abscess.

The patient was urgently transferred to the Department of Neurosurgery in Florence and the abscess was drained after craniotomy. Microbiological examination of the brain abscess specimens was positive for *F Nucleatum*.

Immediately after surgery, the patient underwent a full diagnostic evaluation to identify the potential source of *F Nucleatum*. The chest X‐ray excluded other abdominal or thoracic abscesses. The otolaryngological evaluation was negative on fibroscopic endoscopy. The color Doppler echocardiogram performed to exclude endocardiac vegetation showed a mitral valve prolapse without any endocardiac vegetation. All the main potential sources of infection were ruled out in the first analysis during the hospital stay. In addition, to try to complete the diagnosis, the patient was sent to the regional Center for the Study of Immunodeficiency of the University of Florence, to rule out the presence of specific immunosuppression factors. All tests (Immunoglobulin with subclasses, study of the complement system, and chemotaxis of Polymorphonuclear cell family) were negative. The patient underwent antibiotic therapy (Piperacillin/Tazobactan 2 g/0.25 g ‐ 3 im injection, Amoxicillin/Clavulanic Acid 2 g for 10 days, Metronidazole 400 mg × 3 for 2 weeks), anti‐comitial therapy (Phenobarbital 100 mg/d), and Cortisone therapy (Soldesam 4 mg 1 im injection for 1 week and 2 mg for 1 week).

The patient completely recovered from the neurological disorders and was discharged in November 2009.

As the main sources of infection (abdominal or thoracic abscess, endocardiac vegetation) had been ruled out, the patient was asked specifically to have a periodontal examination. A few months later, in April 2010, the patient went to the Department of Periodontology of the University of Florence for an evaluation.

During an extensive medical interview, the patient reported that he had not undergone a professional oral examination for many years and that he had had a professional oral hygiene session 10 days before the onset of the neurological symptoms. He also remembered copious bleeding during and after the session; no antibiotic therapy was prescribed.

The patients signed a written informed consent with agreement to use his data for the case report, in accordance with the Helsinki declaration of 1975, as revised in 2013.

**Figure 2:**
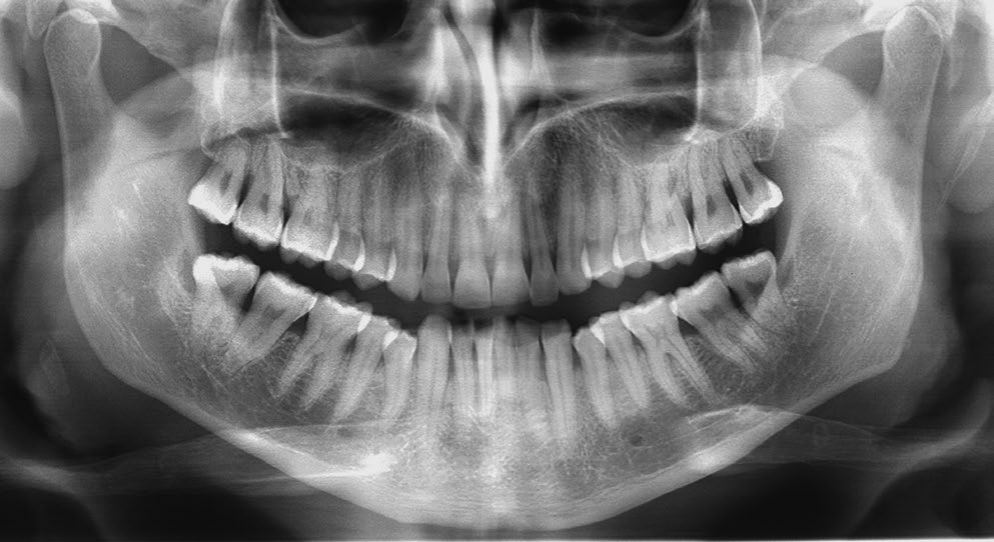
Orthopanoramic X‐ray in 2010 after the onset of the brain abscess

At first visit in the Department of Periodontology of the University of Florence, the patient showed the orthopanoramic X‐ray prescribed by the neurosurgeon (Figure 2).

**Figure 3:**
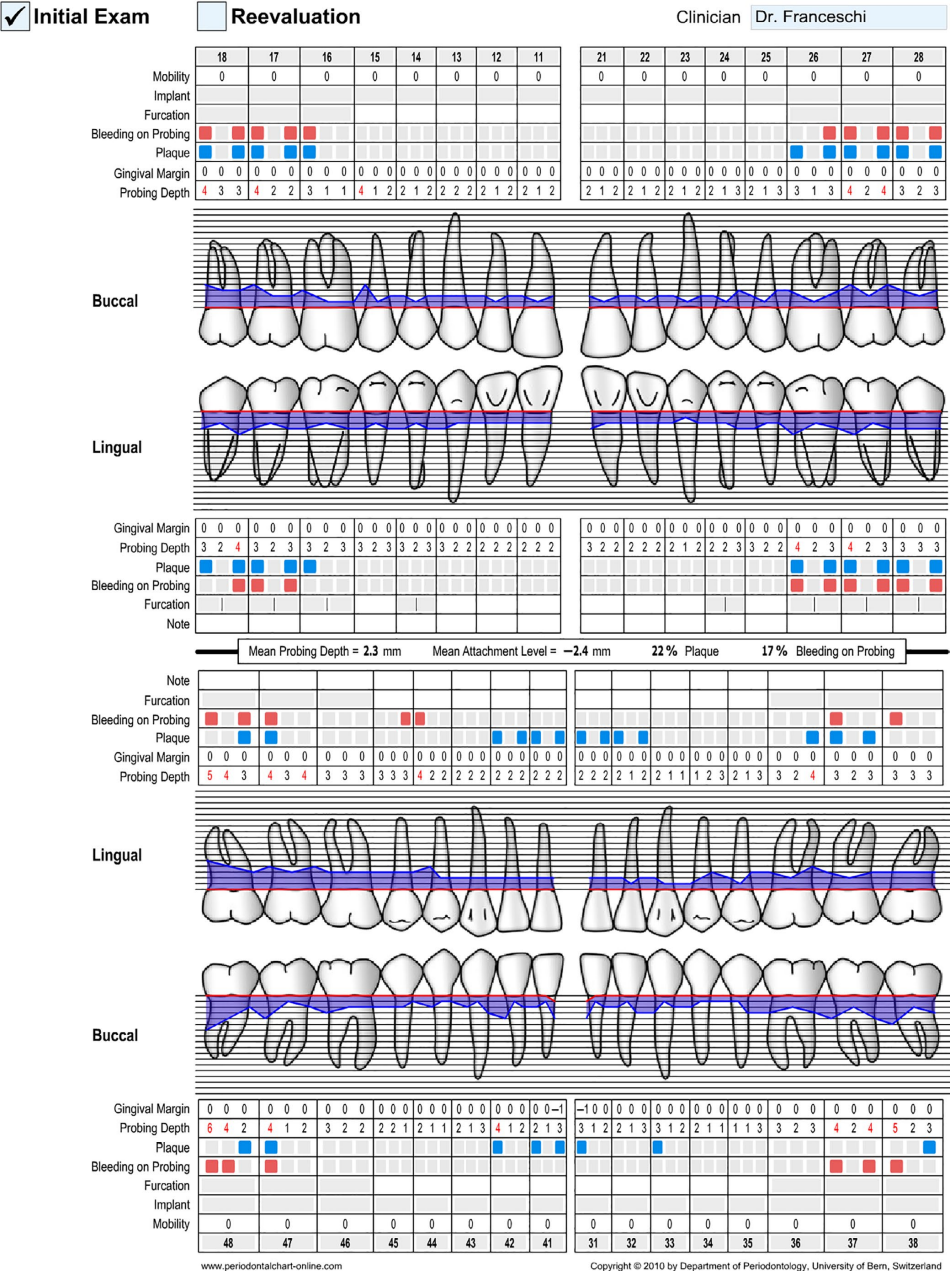
Periodontal Flow Chart of the patient at first evaluation

Periodontal probing depth at six sites per tooth was measured and the periodontal flow chart was drawn up (Figure 3). Only few sites showed moderate probing depth >4 mm (range 4‐6 mm). Basing on the AAP/EFP classification system of periodontal disease,^12^ the patient was classified with a periodontitis Stage II, Grade A.

**Figure 4:**
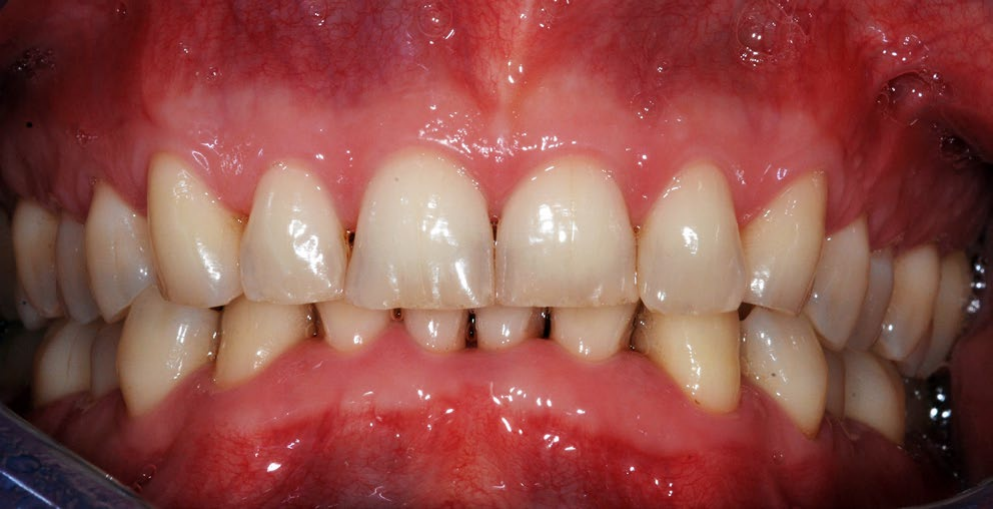
Clinical image of the patient at first visit

The clinical evaluation confirmed a good plaque control (FMPS 22%) (Figure 4).

A real‐time PCR test (Lab*Oral*; LabOral Diagnostics) was performed using the deepest sites: 6 mm in the distal aspect of the right mandibular third molar, 5 mm in the mesial site of the left mandibular third molars, and 4 mm in the mesial aspect of the left maxillary second molar. The results of the microbiological test revealed a significant presence of *F Nucleatum* (5.0 × 10^7^ unit), in the tested sites. (Table 1).

**Table 1:** Microbiological findings of the tested sites in 2010 and 2017

	Microbiological finding	Sites used for the Test and PD
PCR test 2010	*Fusobacterium Nucleatum* (+++)	48 d – 6 mm 38 m – 5 mm 27 d – 4 mm
PCR test 2017	*Parvimonas Micra* (+) *Filifactor alocis* (+)	48 d – 3 mm 38 m – 3 mm 27 d – 3 mm

The patient underwent causal therapy, with antibiotic prophylaxis (2 g Amoxicillin 30 minutes before the treatment) and was given strict oral hygiene instructions.

After 6 weeks, a new periodontal chart was drawn up. The patient had a low plaque index (FMPS 8%) and bleeding on probing (FMBS 7%). All sites showed a probing depth (PD) ≤4 mm, except for two sites (6 mm distal aspect of the third mandibular right molar, 5 mm in the mesial site of the left mandibular third molars). On the basis of the results of perio‐chart, no periodontal surgery was performed.

The extraction of the affected third molars was not planned in order to avoid the potential risk of bacteremia. Therefore, the patient had to follow a strict maintenance program. He had to have professional oral hygiene sessions every 6 months. Each session always showed low Full Mouth Plaque and Full Mouth Bleeding scores. The professional oral hygiene was always performed in association with antibiotic prophylaxis (Amoxicillin 2 g 30 minutes before the session). Probing depth was recorded at each recall visit, showing good periodontal health with no plaque and no periodontal bleeding on probing.

After 7 years, in 2017, the patient's systemic and periodontal health were good. In the tested sites probing depth decreased from 6 mm (distal lower right third molar), 5 mm (mesial lower left third molar) 4 mm (distal upper left second molar) to 3 mm each. Full mouth plaque score was 2% and Full Mouth Bleeding Score was 6%.

A microbiological test in the same previous sites was repeated. The real‐time PCR test (Lab*Oral*; LabOral Diagnostics) was weakly positive for *Parvimonas micra* (+) and *Filifactor alocis* (+). *F Nucleatum* was absent (Table 1).

## 3 DISCUSSION

Brain abscess is a rare and threatening infection caused either by trauma, neurosurgical complication or by secondary infection occurred after bacteremia.

Since brain abscess often occurs spontaneously, the source of infection is searched retrospectively. Often the diagnosis has been one of exclusion as opposed to evidence.

The presence of *F Nucleatum* in the oral cavity has been frequently associated with brain abscesses. Several reviews were published regarding the possible association between brain abscess and oral infections,^1^, ^13^, ^14^ but in most of the cases, it was not possible to establish a true association between intracranial and oral pathogens.

Ewald et al^11^ reported 6 cases with cryptic infections of CNS that occurred probably secondary to oral infection. The results of the microbiological test of the intracranial samples showed the presence of pathogens typically isolated in the oral cavity. Any other microbiological test was performed to confirm the primary oral infection. He proposed three criteria for establishing the diagnosis of odontogenic brain abscess: (a) no alternative source of bacteremia; (b) microbiological finding reveal pathogens typically found in oral microflora; (c) clinical or radiographic signs of active dental or periodontal disease.

Marques Da Silva^15^ described a case of brain abscess caused by *Streptococcus Constellatus* in an immunocompromised patient where periodontal infection was suspected. The brain abscess and periodontal isolates were compared by means of one phenotypic and three genetic fingerprinting techniques. The phenotypic method showed identical profiles between brain and periodontal samples, while ribotyping and RAPD showed very close similarity.

Despite many case reports^11^, ^15^ and literature reviews^1^, ^13^ (Corson et al, 2001) described the possible association between brain abscess and oral infections, a clear relationship between pathogens found in the brain abscess and those of oral origin is lacking.

Melcher et al^16^ published an interesting report on the 2800‐year‐old Egyptian mummy of Djedmaatesankh (*c*. 945‐715 BC) The CT scan of the mummy showed the presence of many teeth exhibiting exposure of dental pulp and periapical lesions. A large cyst has displaced the maxillary antrum and enlarged the maxilla. Pus from the cyst may have drained different sinuses resulting in her death.

Two main considerations can be drawn from this case report.

The present case confirmed the presence of the same microbiota isolated in the brain abscess and those found in periodontal pockets. Unfortunately, the patient was sent for the periodontal evaluation few months after the neurological surgery, so it was not possible to match the genetic analysis of the intra‐ and extracranial sites. If the patient was evaluated few days after the results of the microbiological finding of the brain abscess, it could be reasonable to match genetically the two microbiological findings (intra‐cranial/ oral) to highlight the possible oral origin of the brain abscess. After 1 week, it was not possible to process the brain abscess sample for a genetic analysis.

The patient described in this report is interesting and surprising because he was and is a systemically healthy person with good periodontal health with good plaque control and physiologic probing depths. A diagnosis of moderate periodontitis could be made (Stage II, Grade A). The most important point in the anamnesis was that he reported having copious bleeding during and after a session of oral hygiene. The potential bacteremia probably caused the colonization of the microbiota *F Nucleatum* and the subsequent brain abscess. As the principal sources of other systemic infections including the presence *F Nucleatum* had ruled out, the hypothesis of a periodontal bacteremia causing the brain abscess gains further strength.

It is important to underline that *F Nucleatum* could also be merely an accidental finding as it is not unusual to find presence of this bacteria in periodontal pockets; although all other causes have been excluded, concomitant presence does not necessarily mean cause‐effect relationship.

The second consideration shows that unfortunately, it may be impossible to predict the onset of a brain abscess and the first urgent therapy is neurosurgery to save the patient and to reduce the neurologic symptoms. Immediately after surgery, it is crucial to make a rapid and exact diagnosis. All cases of brain abscess should be evaluated through broad‐scale diagnostics that must include periodontal assessment, especially those cases without any other systemic involvement. Patients treated for brain abscess, with suspect of oral infection should be soon evaluated by a periodontist, to analyze the possible oral origin of the brain abscess. If the matching of the genetic analysis confirms the correlation between the two species, the patient should be treated to avoid new contamination.

This case of a brain abscess developing in a young and systemically healthy patient shows the importance of combined diagnosis involving both medical and dental practitioners, and the essential need for periodontal assessment. The patient was treated and followed for a long period of time, showing, at every recall visit, low plaque score and low bleeding score. The microbiological test performed after 7 years in the same pockets, showed the absence of *Fusiobacterium Nucleatum*.

## 4 CONCLUSIONS

Today, in the era of “Periodontal‐Medicine” approach, close cooperation between physicians and periodontists is very important.

Genetic matching is necessary to verify the true cause/effect relationship between periodontal pathogens and brain abscess. However, all cases of brain abscess should be evaluated through broad‐scale diagnostics that must include periodontal assessment, especially those cases without any other systemic involvement. Patients reporting acute systemic infection such as brain abscess associated with typical periodontal pathogens (FN) must follow an appropriate periodontal therapy and stringent protocol of oral hygiene maintenance.

## CONFLICT OF INTEREST

None declared.

## AUTHOR CONTRIBUTIONS

Franceschi Debora: involved in data curation, investigation (equal), Writing‐original draft preparation, and Writing‐reviewing and editing (equal). Giuliani Valentina: involved in investigation (equal), writing‐reviewing and editing (equal). Giuntini Veronica: involved in writing‐reviewing and editing (equal). Pini Prato Giovan Paolo: involved in supervisor and writing‐reviewing and editing (equal).

## ETHICAL APPROVAL

The study was approved by the Ethical Committee, *Area Biomedica University of Florence* (2019).
